# A comprehensive study on non-governmental actors in shaping grassland ecological compensation within legal frameworks

**DOI:** 10.1038/s41598-024-56146-7

**Published:** 2024-03-06

**Authors:** Ziqi Liu, Jiyao Sun

**Affiliations:** 1 College of Politics and Law, Northeast Normal University, Changchun City, Jilin Province China; 2 School of Marxism, Xidian University, Xi’an City, Shanxi Province China

**Keywords:** Ecological compensation, Non-governmental entities, Environmental governance, Protection of rights and interests, Diversified development, Environmental impact, Grassland ecology

## Abstract

Ecological compensation has emerged as a crucial institutional framework for managing the interplay between ecological preservation and economic development in China. This study focuses on the specific case of grassland ecological compensation to investigate the protection of rights and interests of non-governmental subjects. By utilizing data derived from questionnaire responses, this study examines the legal rights, obligations, and responsibilities associated with grassland ecological compensation. Statistical techniques such as Z-distribution, chi-square test, and non-parametric measures of correlation are employed to analyze the collected data, which are presented using tables and graphs. Furthermore, this research evaluates the current state of rights and interests of compensation subjects engaged in ecological compensation practices, aiming to enhance our comprehension and assessment of the extent to which the ecological compensation system safeguards the rights and interests of individuals. The findings show that a substantial number of respondents see current grassland ecological compensation methods in China as reasonable but insufficient, indicating a need for method diversification. There’s a clear preference for a shared responsibility model over government-only funding, especially in regions with large grassland areas. This highlights the necessity for adaptable laws and a legal framework that accommodates diverse stakeholder needs. Additionally, the importance of clear property rights is emphasized for sustainable land use. The study suggests legislative reform towards a more equitable and effective approach to grassland conservation, providing valuable recommendations for refining and advancing the ecological compensation system.Author name 1 (Ziqi Liu) mismatch between ms and metadata. We have foolowed metadata. Kindly check and confirm.The metadata is right. Thank you.

## Introduction

Ecological compensation serves as an institutional framework^1–3^ for managing the interplay between ecological environment regulation and economic development. While originally imported from the West^4^, it has now become essential for the construction of ecological civilization, green economic transformation, and modernization of national governance in China^5,6^. Over the years of development, ecological compensation has played a crucial role in poverty alleviation, rural revitalization, ecological equity maintenance, and promotion of economic and social development. However, the government’s monolithic model has posed limitations to ecological compensation, leading to issues such as a single source of compensation funds, sluggish operational efficiency, and limited participation of compensation subjects.

The practice of ecological compensation encompasses a series of activities^7^ within the ecological compensation system. It serves as both the starting point and the culmination of ecological compensation, reflecting the guidance and constraints of the system on ecological compensation practice, as well as the response of ecological compensation practice to institutional arrangements. Essentially, the practice involves ecological compensation subjects whose behaviors are influenced and compelled by the system, as well as driven by their pursuit of rights and interests. Consequently, different types of subjects, including individuals, enterprises, and NGOs, exhibit diverse behaviors and express their rights and interests differently within ecological compensation practices. These differences arise from variations in status, organizational forms, and value objectives. Although the rights and interests of compensation subjects have gradually become standardized, democratized, and scientific, they have historically been underrepresented in ecological compensation mechanisms. Their behaviors have not been adequately represented, and the inclusion of compensation subjects^8,9^ within the institutional framework of ecological compensation remains insufficient. Therefore, this study aims to present a comprehensive overview of the rights and interests of subjects involved in ecological compensation practices.Please check the layout of Table(s) 1, 3, 5, 6, and correct if necessary.It is right. Thank you!

To start with, the current legal framework of ecological compensation in China should be introduced to identify current compensation gaps. The current legal framework of ecological compensation in China primarily revolves around the concept of “ecological civilization” and is governed by a set of laws and regulations aimed at promoting environmental protection and sustainable development. One of the key legal documents in this regard is the Environmental Protection Law of the People’s Republic of China, which emphasizes the principle of “polluter pays” and lays down the foundation for ecological compensation mechanisms. Additionally, *China’s Forest Law and Grassland Law* provide further provisions for the protection and restoration of ecosystems, contributing to the framework for ecological compensation^10^. Furthermore, recent initiatives such as the “*Ecological Compensation Mechanism Implementation Plan*” issued by the State Council in 2016 underscore the government’s commitment to establishing comprehensive mechanisms for ecological compensation nationwide^11^. Notably, specific attention is given to the protection and restoration of grassland ecosystems, which are vital components of the country’s natural environment. The *Grassland Law of the People’s Republic of China* outlines measures for the conservation and sustainable management of grasslands, recognizing their importance for biodiversity, carbon sequestration, and pastoral livelihoods^12^. To address degradation and promote restoration, various compensation mechanisms have been established, including subsidies for grassland protection, restoration projects, and payments for ecosystem services provided by pastoralists. These initiatives aim to incentivize sustainable land management practices, such as rotational grazing and reseeding, while also providing economic support to rural communities dependent on grassland resources. Furthermore, pilot programs and local regulations have been implemented in regions such as Inner Mongolia and Qinghai Province to tailor compensation schemes to the specific needs and challenges of grassland ecosystems. By integrating grassland ecosystem compensation into the broader legal framework for ecological conservation, China endeavors to ensure the long-term resilience and sustainability of its grassland landscapes.Please confirm the section headings are correctly identified.Yes. But there are two *Supporting Information* headings. Please check.

These legal instruments provide the necessary framework for implementing ecological compensation practices, ensuring that entities responsible for environmental degradation bear the costs of restoration and conservation efforts. Nonetheless, the legal system falls short in fully taking account the right of compensation subjects.

Firstly, there’s a lack of comprehensive representation and inclusion of all relevant stakeholders, including local communities, indigenous peoples, and marginalized groups, in decision-making processes related to compensation allocation and implementation. This exclusion can lead to disparities in the distribution of benefits and may exacerbate social inequalities^13^. Secondly, while efforts have been made to standardize and democratize the rights and interests of compensation subjects, significant gaps remain in ensuring equitable access to compensation mechanisms, particularly for vulnerable populations with limited resources or legal knowledge^4^. Moreover, the scientific basis for determining compensation amounts and methodologies may be inadequate, leading to inconsistencies and uncertainties in assessing the true value of ecosystem services and the extent of environmental damages^14^. Without robust scientific methodologies and data, there is a risk of undervaluing or overlooking the ecological contributions of compensation subjects, which can undermine the effectiveness of compensation schemes in achieving environmental objectives. Additionally, enforcement mechanisms and monitoring systems for compliance with compensation agreements are often weak or lacking, resulting in instances of non-compliance or inadequate implementation by compensation subjects. Overall, addressing these insufficiencies requires a holistic approach that prioritizes stakeholder engagement, equitable access to compensation mechanisms, improved scientific methodologies for valuation, and strengthened enforcement and monitoring systems to ensure accountability and effectiveness in ecological compensation practices.

In the case of China, the grassland ecosystem, which distributed across the north, northeast, Qinghai-Tibet, and south regions, not only interacts with other ecosystems but also holds significant economic and cultural value, particularly due to unique production methods and the presence of ethnic minorities. The author’s involvement in the research project “*Grassland ecological compensation legal system construction under the perspective of pastoral ecological civilization*” provided ample first-hand data through research conducted in five provinces: Inner Mongolia Autonomous Region, Sichuan Province, Qinghai Province, Gansu Province, and Jilin Province. Through careful analysis and comprehensive summaries of this research data, a more objective portrayal of the current situation of compensation subjects’ rights and interests in the compensation practice can be presented.

Specifically, our exploration mainly considers the following research questions:

1. Does the level of support for the legalization of grassland ecological compensation vary among respondents from different occupational backgrounds?
2. Do compensation subjects within the study area perceive the methods of ecological compensation as reasonable and effective in addressing the needs of grassland conservation?
3. How should funds for grassland ecological compensation be allocated, and is it appropriate for this to be the sole responsibility of the government?

## Theoretical framework

In the academic realm, the concept and content of ecosystem compensation are widely debated^15–20^. Some scholars argue for a government-led approach to diversified ecological compensation, with other subjects and the market playing subsidiary roles, while others advocate for a market-led approach with equal participation from the government and other subjects. Establishing an overall consensus is crucial before conducting research on specific issues, such as the protection of the rights of non-government subjects^21^. Moreover, the study of “pluralistic subjects” requires further exploration. Understanding how non-governmental participants can become key subjects in pluralistic ecological compensation^22,23^ is a fundamental question that needs to be addressed. However, existing research predominantly focuses on the plurality of subjects without adequately investigating this core issue^24–27^. Consequently, non-government subjects have been overlooked in the development of ecological compensation pluralism^8,9,21^, and the variety of approaches and methods based on multiple subjects have struggled to escape the constraints of formalism. Furthermore, there is a scarcity of research from the perspective of rights and interests of compensation subjects in the realm of ecological compensation. Existing studies primarily concentrate on the phenomena and key issues of diversified ecological compensation, such as compensation subjects, methods, and sources, while neglecting the protection of participating subjects’ rights, particularly non-governmental subjects engaged in ecological compensation on an equal footing.

The emergence of research on non-governmental subjects in the field of public administration has been relatively recent, focusing on administrative aspects. Some studies^8,9^ highlight the characteristics of non-government subjects in terms of their service-oriented nature, non-power-based approach, decentralized nature, and organizational diversity. Others^6,21,28^ emphasize the effectiveness of non-governmental participation in urban public crisis management through the establishment of crisis culture, dedicated crisis management bodies, and clarification of responsibilities, powers, and benefits of non-governmental subjects. Similarly, in the context of government land violations, studies analyze the theoretical basis for illegal actions by local governments and governance approaches based on different needs, behavioral motivations, and governance instruments. Additionally, in grassroots social governance^4,27,29^, research explores the collaboration of multiple actors through co-construction, co-management, and resource sharing. In the realm of carbon–neutral governance, studies^15,16,28,30,31^ address the power-rights imbalance between the government, enterprises, and the public, and propose a “trinity” governance approach encompassing government-led governance, enterprise responsibility, and active public participation. However, few studies have examined the legal system of ecological compensation or grassland ecological compensation. Existing research either focuses on compensation for factors other than grasslands or solely examines specific elements within the grassland compensation policy^7,32–34^, such as the attainment of compensation targets or the reasonableness of compensation standards. Consequently, a national study that specifically addresses the status and construction of the grassland ecological compensation legal system is lacking.

To fill these research gap, this research will put the focus on grassland compensation subjects. To begin with, the theory of ecological compensation should be clarified. The concept is often traced back to the Coase Theorem^35^, which underscores that ecological resources are a vital public good characterized by certain properties. The exploitation of these resources invariably results in externalities, necessitating mechanisms for compensation and mitigation. Several scholars have contributed valuable insights into this field. Specifically, a study on Ecuador’s water trust funds, which aligns with the Chinese model’s embrace of market mechanisms, reinforces the assertion of diversification in compensation methods, emphasizing the role of innovative financing mechanisms in bolstering ecological conservation endeavors^38^. Muradian et al. (2010) offer a conceptual framework that converges with the Chinese model of relying on a coalition of government, market, and social organizations. Their reconciliatory approach between theory and practice illuminates the intricate dance of these diverse actors in effecting meaningful and sustainable ecological compensation^39^. These works explore the complex interplay between economic and ecological systems and highlight the importance of equitable exchanges between these realms. Professor Tacconi^36^, for example, defines ecological compensation as conditional payments made by beneficiaries for ecological services tailored to meet specific needs, which contributes a perspective that resonates with the Chinese discourse, redefining payments for environmental services to accommodate broader and more integrated applications. This redefinition aligns with China’s model of diversifying ecological compensation subjects, encapsulating government, market, and societal forces in a cohesive framework. Despite varied perspectives on the concept, a common thread among scholars is the emphasis on exchanging material or economic benefits for ecological resources and services.

On the basis of the above-mentioned consensus, the research tendency and trend of ecological compensation models in academia has completed two changes from government-led compensation models, to market-led compensation models, and finally to compensation models combining government and market. Besides, the theory of diversified models has begun to receive more and more attention and is gradually recognised and accepted. In practice, due to the diversity of ecological resources, countries often choose their own ecological compensation models according to their specific national conditions and needs. For example, trust funds and equity financing for ecological enterprises in the US, Ecuador and Costa Rica have formed more mature models and accumulated rich experience in actual operation. In general, the trend is to move from government-led to fully market-based development. The current choices of ecological compensation models in the world are as follows: In Japan, a government-led approach involves establishing a “forest environment preservation fund” funded by downstream water users to compensate those in upstream water catchment areas for safeguarding their water sources. Meanwhile, the United States follows a market-led model with wetland mitigation banks, where wetland credits are generated by restoring wetlands and sold to interested parties for development. France utilizes a quasi-market transaction model for ecological compensation agreements between downstream water farmers and upstream mineral water plants. Ecuador employs a combined government and market model through a water fund created by NGOs and government authorities to incentivize upper Pafon River farmers to protect their water sources. Costa Rica adopts a public–private partnership model with a payment program for forest environmental services, while Zimbabwe implements a public–private partnership project in collaboration with rural self-governments and impoverished communities to safeguard ecosystems and wildlife. These diverse models demonstrate various approaches to ecological compensation worldwide.

**Figure 1:**
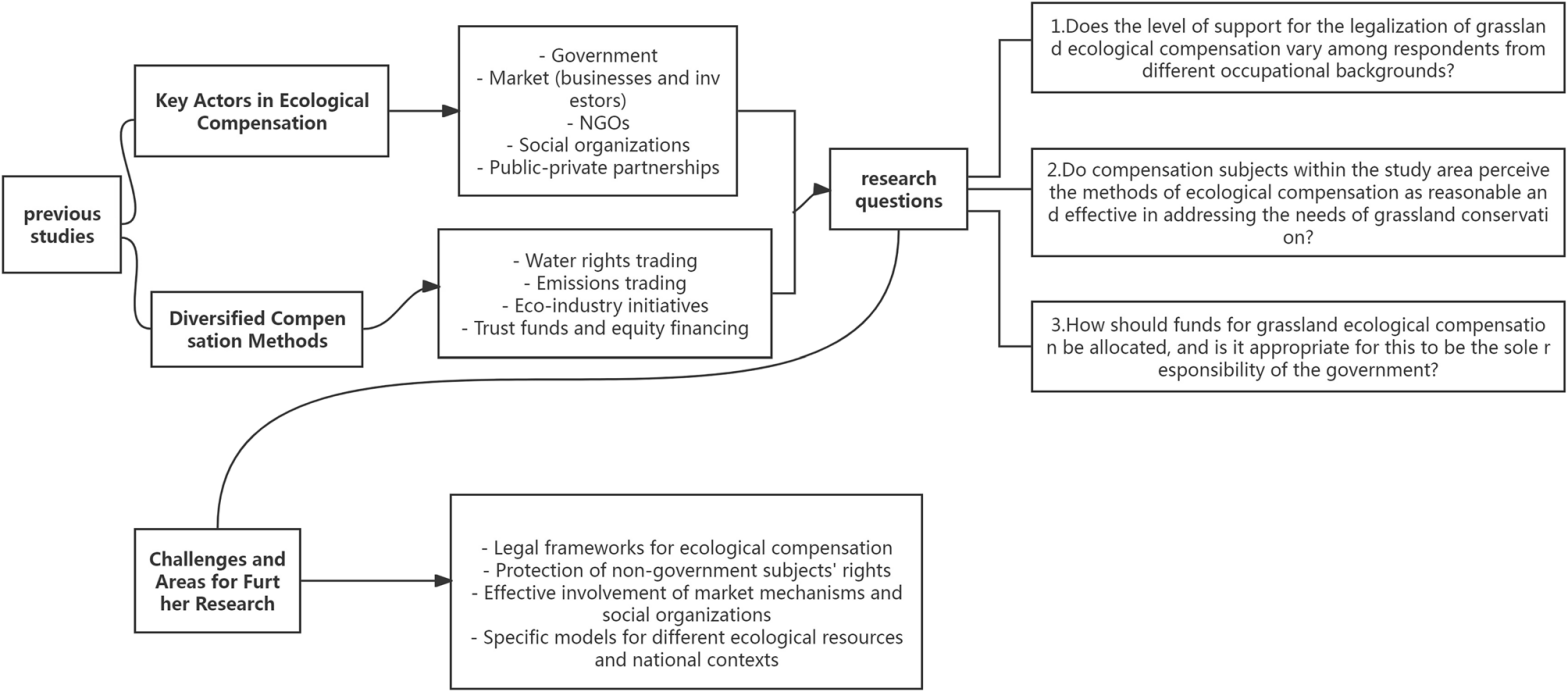
Theoretical framework.

The theory of diversified ecological compensation in China has been widely researched, and scholars have put forward its concept and framework from different perspectives, arguing that its essence lies in relying on multiple subjects to jointly implement multi-channel compensation and improve the efficiency of ecological compensation^37,38^. The development model of diversified ecological compensation is mainly reflected in the diversification of the composition of subjects and compensation methods, with the composition of subjects focusing on the participation of government, market and social organizations^8,21,31,39^, while the diversification of compensation methods mainly reflects the introduction of market mechanisms^28,40,41^. The diversified forms of ecological compensation include water rights trading, emissions trading and eco-industry^5,6^, reflecting both the market mechanism and the role of government and social organizations. The practice and theoretical research of China’s diversified ecological compensation pursue the combination of government, market and social forces, but in practice, market mechanisms and social organisations are still not sufficiently involved, which is also the direction of development^22,29,42^. In general, theoretical research on diversified ecological compensation in China is relatively rich, but practice is relatively backward, and needs to be explored in depth in terms of institutions, implementation paths and cases^30,31^. For a clearer presentation, the framework for this research is presented in Fig. 1.

## Method

Based on objective conditions and statistical principles, the research team chose to conduct a sample survey. Participant informed consent was obtained prior to conducting this study. The research protocol and procedures adhered to ethical guidelines and were approved by the ethics committee of of the School of Politics and Law, Northeast Normal University (Approval No. 201922). All methods in this study were performed in accordance with the relevant guidelines and regulations given by this committee. All participants were provided with detailed information about the study objectives, procedures, and potential risks or benefits involved. They were assured of their right to voluntary participation, the confidentiality of their responses, and the use of data solely for research purposes. By completing and returning the questionnaires or participating in face-to-face interviews, participants indicated their informed consent to be part of the study. No personal identifying information was collected to ensure anonymity and confidentiality. Participation in the research was voluntary, and participants had the right to withdraw from the study at any time without any adverse consequences.

In the sample survey, the selected provinces hold significance due to their large land areas and their inclusion among the 13 key provinces implementing the “grassland subsidy” policy. These provinces span across the northeast, northwest, and south regions, offering broad regional representativeness. Specifically, Inner Mongolia Autonomous Region, Sichuan Province, Qinghai Province, Gansu Province, and Jilin Province rank among the top 10 provinces in China in terms of total grassland area or natural grassland area per capita. Moreover, Inner Mongolia Autonomous Region, Qinghai Province, and Gansu Province are key areas for implementing the subsidy policy, with Inner Mongolia Autonomous Region ranking first in the country in terms of both total area and total subsidy amount. Qinghai Province ranks third in the country in terms of subsidy amount. Furthermore, the selected provinces and regions continue to serve as key experimental areas in the “new round of grassland subsidy policy” implemented in 2016. Geographically, the chosen provinces and regions cover the northeast, northwest, and south, providing a more representative coverage.

Regarding the selection of survey respondents, the study focused on farmers, herdsmen, and grassroots grassland managers who are central to the grassland ecological compensation legal system and the implementation of the grassland compensation policy. This approach aimed to accurately reflect the understanding, opinions, suggestions, and evaluations of farmers, herdsmen, and grassroots grassland managers regarding the “grassland compensation” policy, as well as to capture public opinion trends related to the construction of the grassland ecological compensation legal system.

### Ethics approval and consent to participate

The research protocol and procedures adhered to ethical guidelines and were approved by the ethics committee of of the School of Politics and Law, Northeast Normal University (Approval No. 201922).

## Data and analysis

### Questionnaire survey

This study scrutinizes six pivotal issues pertaining to grassland ecological compensation in China, including: the degradation of family grass pastures^43^; the legalization of grassland ecological compensation^44^; the rationality of the compensation method; compensation distribution; the funding source of grassland ecological compensation^45^; and relevant Institutional oversight.

There are 24 questions in the questionnaire (see Online Appendix I), 11 questions in the first part, including 8 single-choice questions and 3 multiple-choice questions; 5 questions in the second part, including 4 single-choice questions and 1 multiple-choice question; and 8 questions in the third part, all of which are single-choice questions. The questionnaire was designed through six processes: consultation with data users and potential respondents; reference to other questionnaires; drafting of questions; discussion and revision of the questionnaire; pre-placement and revision of the questionnaire; and finalisation. The main methods of analysis of the questionnaire were the estimation of the overall proportion through the sample using the Z distribution, the chi-square test in non-parametric testing methods, and non-parametric measures of correlation.

Considering the distribution format of this questionnaire, which was distributed along with the survey, as well as the language differences and education levels of the respondents (most of whom were herders), the questionnaire was designed with the principle of respecting ethnic diversity and asking for facts before asking for attitudes and intentions, and the Mongolian version of the questionnaire was translated for the areas where Mongolians live. In the basic information section of the questionnaire, respondents were asked about their age, education level, average annual household income, area of grass pasture and degree of degradation of grass pasture. These basic statistics will be combined with the specific questions on the Grassland Grant policy to draw conclusions.

To conduct the study, a total of 8 primary sampling sites, 12 secondary sampling sites, and 10 survey sites in cities, counties, and townships were established based on administrative districts. In total, 479 questionnaires were distributed, and 476 valid ones were collected for analysis. Out of the 476 collected questionnaires, 280 were from farmers and herdsmen, accounting for 58.8% of the total, while 196 were from grassland managers, accounting for 41.2% (refer to Table 1).

**Table 1:** Occupational composition of survey respondent.

	Herders	Grassland managers	Total
Research area			
Inner Mongolia			
Headcount	134	137	271
Percentage	49.4%	50.6%	100.0%
Gansu			
Headcount	16	34	50
Percentage	32.0%	68.0%	100.0%
Qinghai			
Headcount	53	19	72
Percentage	73.6%	26.4%	100.0%
Jilin			
Headcount	23	6	29
Percentage	79.3%	20.7%	100.0%
Sichuan			
Headcount	54	0	54
Percentage	100.0%	0.0%	100.0%
Total			
Headcount	280	196	476
Percentage	58.8%	41.2%	100.0%

#### Degradation of family grass pastures

According to the statistics of the returned questionnaires, 387 people filled in the degree of degradation of the family grass pasture, accounting for 81.3% of the total number of questionnaires. Only 38 people (8.0%) thought that the family grass pastures were not degraded, 146 (30.7%) thought that the family grass pastures were mildly degraded, 145 (30.5%) thought that the family grass pastures were moderately degraded, and 58 (12.2%) thought that the family grass pastures were severely degraded (see Table 2).

**Table 2:** Extent of degradation of household grass pastures of survey respondents.

	Frequency (households)	Percentage (%)	Cumulative percentage
Severe degradation	58	12.2	12.2
Moderate degradation	145	30.5	42.6
Mild degeneration	146	30.7	73.3
Undegraded	38	8.0	81.3
Not filled	89	18.7	100.0
Total	476	100.0	

**Figure 2:**
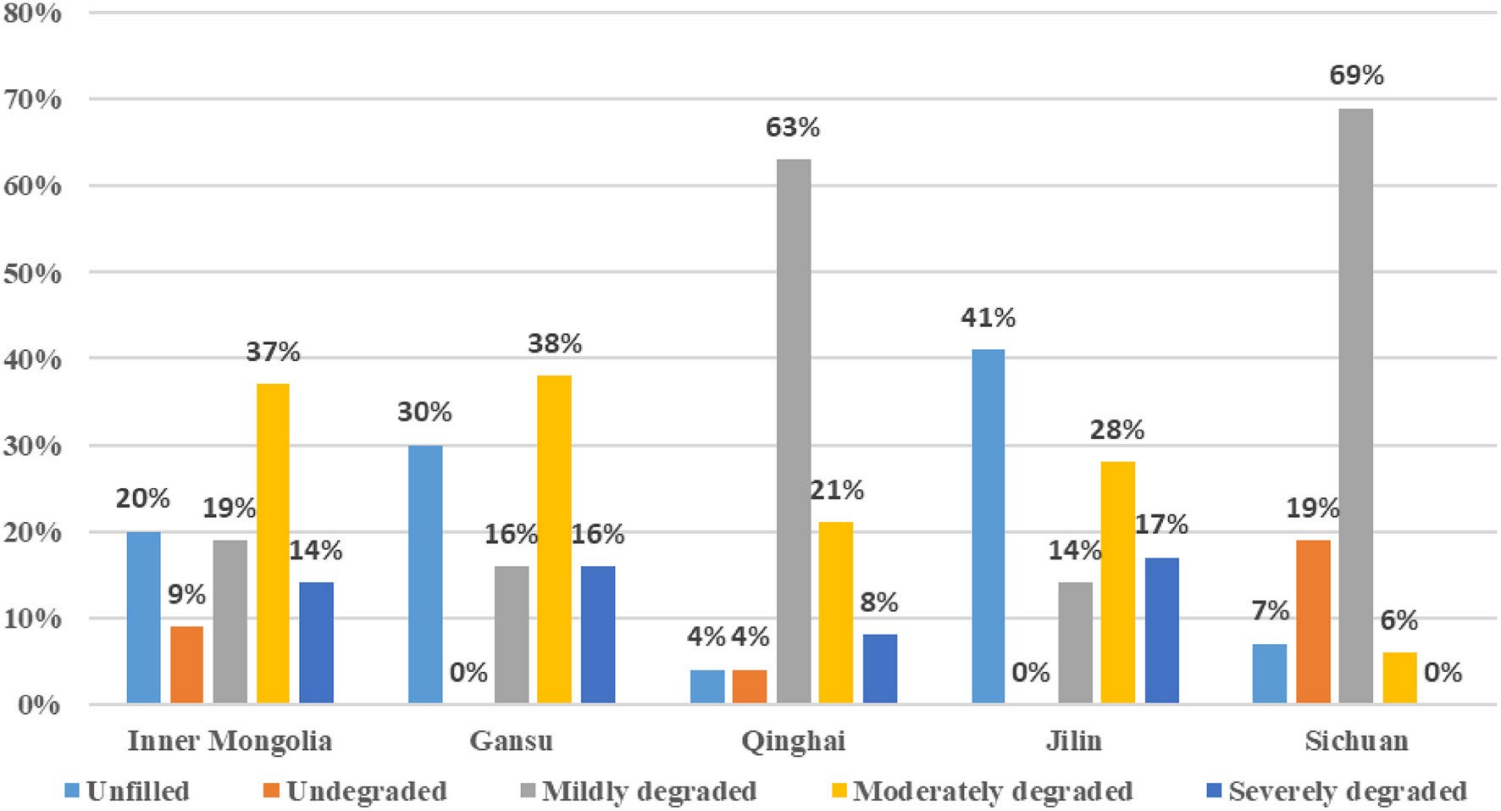
The extent of degradation of household grass pastures by study area.

In general, the degradation of family grass pastures in China is quite common, mostly at the level of mild and moderate degradation, with severe degradation accounting for a small proportion. Based on a comparison of the degree of degradation of family grass pastures by province, 37% and 38% of family grass pastures in Inner Mongolia and Gansu Province respectively were mainly moderately degraded, while 63% and 69% of family grass pastures in Qinghai and Sichuan Provinces were mainly lightly degraded respectively. (See Fig. 2).

#### The legalization of grassland ecological compensation

In the first part of the questionnaire, questions 1 and 4 were statistically analysed using two methodologies: Z-distribution for sample estimation of the overall proportion and the chi-square test as a non-parametric test. This study used a sample of 476 respondents comprised of herders and grassland managers. The confidence interval for the overall proportion of each option was estimated, and the chi-square test was utilized to scrutinize the influence of factors such as occupation and education level on the inclination towards the legalization of grassland ecological compensation.

Regarding the legalisation of grassland ecological compensation, a considerable majority of the respondents (455 or 95.6%) favoured this notion, whereas a minimal portion of the sample (21 or 4.4%) objected to it.

We then explored if the attitude of support for the legalisation varies between respondents of different occupations. Given the number of herders and grassland managers who favoured legalisation, the standard error of the proportion was calculated according to the formula given by^46^, which was

1 $$S_{p} = \sqrt {\frac{{P\left( {1 - P} \right)}}{N}}$$

where $Sp$ is the standard error of proportion (an estimate of the standard deviation of the sampling distribution of proportions). $p$ is the proportion of the sample whose different occupational choices should be legalized options, 93.9% for herders and 98.0% for grassland managers (see Table 3). $n$ is the total number of the sample, 280 herders and 196 grassland managers, respectively, for a total of 476 people.

**Table 3:** Cross-tabulation of whether grassland ecological compensation should be legalised based on occupation.

	Occupation		Total
	Herders	Grassland managers	
Should it be legalised			
Yes			
Headcount	263	192	455
Percentage	93.9%	98.0%	95.6%
No			
Headcount	17	4	21
Percentage	6.1%	2.0%	4.4%
Total	280	196	476

To investigated whether different occupations influence attitudes towards the legalization of grassland ecological compensation, we conducted a chi-square test using IBM SPSS Statistics 25, involving a 2 × 2 cross-tabulation of respondents’ occupations and their stance on legalizing grassland ecological compensation. The null hypothesis posited no difference in attitudes across occupations, while the research hypothesis suggested a variation. The chi-square test results were as follows: Pearson chi-square value (χ^2^) of 4.442, a degree of freedom (d_f_) of 1, and a significance level (α) set at 0.05. The two-sided asymptotic significance (*p*-value) was 0.035. Given that the critical chi-square value at α = 0.05 and d_f_ = 1 is 3.841, our test value of 4.442 exceeds this threshold, and the *p*-value of 0.035 is less than the significance level of 0.05. Thus, the null hypothesis is rejected, supporting the research hypothesis that occupation significantly influences attitudes towards the legalization of grassland ecological compensation.

For brevity, it’s important to mention that the data analysis section consistently employs the chi-square test to examine opinion differences across various occupations. Detailed procedures are omitted to conserve word count.

#### Exploring the most effective compensation method

We first explored whether grassland ecological compensation methods are reasonable in each research area. The results are as follows: 61% of the respondents believe that the current grassland ecological compensation method is basically reasonable, while 39% believe that it is not reasonable and should be diversified. This indicates that while most herders are satisfied with the current grassland ecological compensation method, a notable number of respondents express dissatisfaction. They critique the method’s homogeneity, primarily monetary in nature, and advocate for a more diversified approach to compensation.

**Figure 3:**
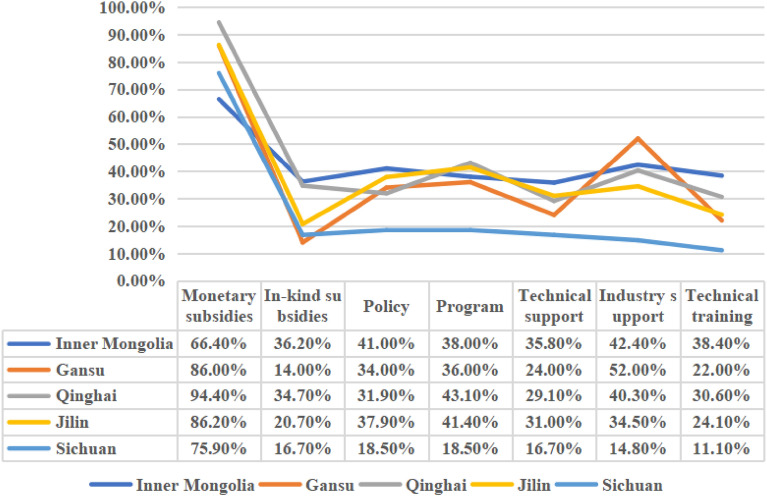
Statistics on the choice of “grassland ecological compensation methods” in each study area.

To determine the most effective compensation method, we conducted a multiple-choice survey. Table 4 categorizes the results by compensation methods. It reveals that monetary compensation was deemed most effective, with 357 respondents (75%) favoring it. Additionally, over 30% of respondents selected other forms of compensation. For a clearer understanding of these preferences across different study areas, we provide a visual representation in Fig. 3.

**Table 4:** Statistics on “Which is the more effective way to compensate grassland ecology” (persons).

Options	Inner Mongolia	Sichuan	Qinghai	Gansu	Jilin	Total	Percentage (%)
Monetary compensation	180	41	68	43	25	357	75.0
In-kind subsidies	98	9	25	7	6	145	30.5
Policy	111	10	23	17	11	172	36.1
Projects	103	10	31	18	12	174	36.6
Technical support	97	9	21	12	9	148	31.1
Industry support	115	8	29	26	10	188	39.5
Technical training	104	6	22	11	7	150	31.5

#### Grassland ecological compensation distribution

On the question of how the grassland ecological compensation funds should be distributed, we present Table 5 for a clear illustration of the research results. It reveals a preference for the one-time fixed amount distribution method, chosen by 253 respondents (53.2% of total responses). The periodic reward and punishment variable distribution method was selected by 223 respondents (46.8%). Breaking it down by occupation, 64.6% of herders favored the one-off fixed compensation, compared to 36.7% of grassland managers. Conversely, 35.4% of herders and 63.3% of grassland managers supported the variable periodic rewards and punishments. Overall, grassland managers tend to support periodic payments, while herders prefer one-off payments.

**Table 5:** Cross tabulation of how occupational and grassland ecological compensation funds are disbursed.

	How compensation funds are disbursed		Total
	One-off payment	Periodic rewards	
Occupation			
Herders			
Headcount	181	99	280
Percentage	64.6%	35.4%	100.0%
Grassland managers			
Headcount	72	124	196
Percentage	36.7%	63.3%	100.0%
Total			
Headcount	253	223	476
Percentage of total	53.2%	46.8%	100.0%

#### Funding source of grassland ecological compensation

In the survey on the source of grassland ecological compensation funds, 41.8% of respondents favored sole government responsibility, while 58.2% supported a combined contribution from the government, society, market, and individuals concerned. This suggests a recognition among most respondents, particularly herders, that government financing alone is inadequate for the substantial compensation demands in grassland pastoral areas. They acknowledge the need for diversified funding sources.

**Figure 4:**
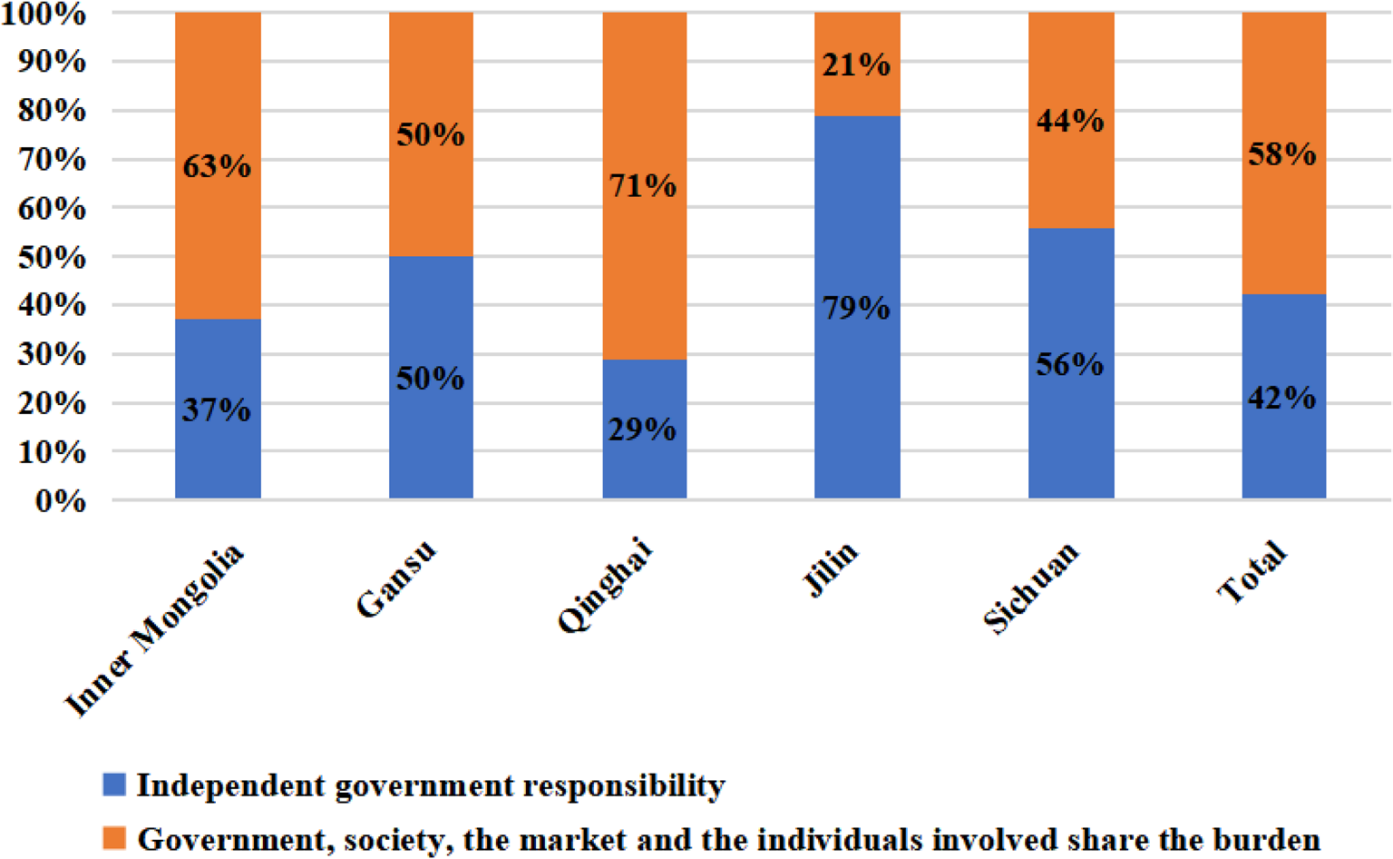
Statistics on the choice of “Where should grassland ecological compensation funds come from” by research region.

Figure 4 details the preferences for compensation fund sources across different research regions, highlighting variations influenced by each province’s unique grassland conditions and socio-economic development. Notably, respondents from Jilin and Sichuan showed higher support for the government bearing the cost alone, at 79% and 56%, respectively. This preference is linked to the specific business models in these regions. Here, enterprises involved in grassland protection often incur losses due to contractual issues when receiving compensation, which underpins the prevalent opinion favoring exclusive government responsibility for compensation funds.

#### Institutional oversight of grassland ecological compensation

We also explored whether a special body should be set up to regulate and supervise the issue of grassland ecological compensation and relief, with the results presented in Table 6**.** The data is segmented into two occupational groups: herders and grassland managers. Among herders, a significant majority (86.4%, or 242 individuals) support the idea of a dedicated relief unit, while only 13.6% (38 individuals) oppose it. The support is even stronger among grassland managers, with 91.3% (179 individuals) in favor and a mere 8.7% (17 individuals) against. Overall, combining both groups, the table shows a clear consensus in favor of a specific relief sector, with 88.4% (421 individuals) supporting the proposal and only 11.6% (55 individuals) opposing it.

**Table 6:** Cross tabulation of whether there should be a specific relief sector for occupational and grassland ecological compensation.

	Whether there should be a dedicated relief unit		Total
	Should	Should not	
Occupation			
Herders			
Headcount	242	38	280
Percentage	86.4%	13.6%	100.0%
Grassland managers			
Headcount	179	17	196
Percentage	91.3%	8.7%	100.0%
Total			
Headcount	421	55	476
Percentage of total	88.4%	11.6%	100.0%

The data first highlights the current lack of adequate rights relief in grassland ecological compensation. This absence is significant, indicating that there is no specialized entity effectively addressing or restraining issues related to these rights. Besides, the data also underscores the urgency and necessity of focusing on rights relief within grassland ecological compensation. The strong support for a dedicated relief unit reflects a widespread recognition among both herders and grassland managers of the immediate need to address this issue. Consequently, the findings suggest an urgent need to establish a comprehensive legal framework for grassland ecological compensation. Such a system would not only ensure the protection and enforcement of compensation rights but also provide a structured approach to supervise and manage these rights effectively. This legal system would play a crucial role in addressing the current gaps and ensuring more equitable and effective management of grassland ecological resources and compensation.

## Discussion

Ecological compensation is pivotal in balancing ecological preservation with economic growth, especially in China’s journey towards green economic transformation and modernized governance. However, the prevailing government-centric model faces challenges like a singular funding source, low efficiency in compensation, and limited stakeholder engagement. Policy analysis and case studies are key to understanding and improving these systems. China’s venture into diverse ecological compensation methods is relatively new, and its practical application is still evolving. Compared to countries with more established practices, China has less experience and faces unique challenges and conditions. In the context of its socialist market economy, relying solely on market mechanisms for ecological compensation is inadequate. Instead, a comprehensive approach involving multiple stakeholders is crucial for the effective implementation of these systems.

The current study explores the role of non-governmental entities in multi-faceted ecological compensation, particularly focusing on the grassland ecological compensation legal system. This discussion emerges in the backdrop of noticeable degradation of family grass pastures in China, with a preponderance of the degradation leaning towards mild to moderate, while severe degradation accounts for a minor segment^47^. Considering the sample estimates, an overall percentage of 54.9–66.5% have expressed that the current approach to grassland ecological compensation in China is basically reasonable. Conversely, 33.5–45.1% view the current method as less than reasonable. This suggests a window of opportunity for diversification and improvement in the grassland ecological compensation methodologies currently in place. The existing legislative mechanisms and laws appear to fall short in addressing the extent and intensity of grassland degradation. Considering that most of this degradation is mild to moderate, this signals an opportunity to intervene and halt this process through effective legislative reforms and enforcement^47^. Besides, the legal system should consider these diverse perspectives when devising reforms, ensuring that the amended legislation reflects the sentiments and expectations of all stakeholders.

The chi-square test results have brought to light differences in opinions among different occupations regarding the reasonable distribution of grassland ecological compensation funds. Herders and grassland managers significantly differ in their preferences; 64.6% of herders advocate for a one-time fixed amount of compensation, whereas 63.3% of grassland managers are inclined towards variable periodic rewards and punishments. These results accentuate the divergence in perceptions of the two groups towards the distribution of compensation funds. It seems that the present legal approach may not be accommodating these nuances adequately. As the law presently stands, there appears to be a one-size-fits-all strategy regarding compensation funds’ distribution. The law must be tailored to recognize and accommodate the different needs and preferences of various stakeholder groups.

An important aspect of this study is the assessment of who should bear the burden of the overall compensation funds. With 99% confidence, the estimates suggest that 36.0–47.7% of the respondents favour the government shouldering this burden independently, while 52.3–64.0% support the collective contribution from the government, society, market, and relevant individuals. The implication here is two-fold. First, the financial responsibility of grassland ecological compensation, which solely rests upon the government currently, is not deemed adequate in fulfilling the extensive demand for compensation funds in grassland pastoral areas. Second, it signifies the varied preferences for sources of compensation funds in different provinces, owing to the distinct natural conditions of grasslands and socio-economic development. In provinces Inner Mongolia and Qinghai, there’s higher support (63% and 71% respectively) for shared responsibility, due to the large per capita grassland area and the constraints of government funding when depending on grassland area for compensation. In contrast, provinces Jilin and Sichuan showed greater inclination towards the government’s independent responsibility for compensation, with 79% and 56% respectively, reflecting the unique regional and business model dynamics^48^.The fact that this shared responsibility approach is particularly favored in regions with large grassland areas per capita suggests the limitations of government funding alone. It is essential for the law to reflect the practical realities of resource availability and allocation. Hence, laws need to be flexible and adaptive, allowing for collaborative and diversified funding strategies where necessary^49^.

Moreover, this research presents comprehensive field investigations and interviews on grassland ownership and ecological compensation in various regions in China. These findings parallel broader academic discourses concerning property rights and environmental policy, shedding light on the complex challenges that are encountered in practice. One key issue identified in the interview is the clarity of grassland ownership. For instance, *the Grassland Supervision Bureau of Banner* reveals that the tenure system has been implemented at the household level, leading to a general understanding of grassland tenure. Nevertheless, there are persistent issues with demarcating boundaries between different zones. This issue mirrors global concerns about property rights, specifically land rights. Clear and enforceable property rights have been advocated as a fundamental condition for efficient and sustainable land use^50^. Thus, a potential policy implication could be to leverage technology, such as Geographic Information Systems (GIS), to accurately map and communicate grassland boundaries.As References [4, 48] and [14, 49] are same, we have deleted the duplicate reference and renumbered accordingly. Please check and confirm.Yes. Thank you.

In the context of Gansu Province, the vastness and diversity of its grassland regions present considerable challenges in census-taking and rights confirmation. Study^51^ stresses the importance of formalizing property rights to bring assets into the formal economy. A tailored, region-specific approach may be needed to clarify grassland ownership, considering the unique characteristics of each area, such as the Qinghai-Tibet Plateau, the western desert, and the Loess Plateau. A study^52^ argued that a range of rights should be taken into account, including exclusion and management, suggesting the need for legal measures that acknowledge and protect a diverse set of rights for different stakeholders.

As for grassland ecological compensation, the concerns raised by herders echo existing literature on environmental compensation and justice. The current compensation rate, according to herders, inadequately addresses all costs associated with protecting the grassland. Further, the standardised compensation approach seems to exacerbate regional and household disparities, arguably leading to social inequality. As studies^53,54^ have noted, environmental policies should not only address ecological concerns but also aim for equity and social justice. Thus, future policy revisions might need to reconsider compensation rates and the methodology for distributing compensation to address these concerns.

## Conclusions

This research explored the support for the legalization of grassland ecological compensation across different occupational backgrounds, the perceived effectiveness of current ecological compensation methods, and the debate surrounding the allocation of compensation funds, including the role of non-governmental entities.

The findings reveal a significant portion of respondents believe the current methods of ecological compensation are reasonable, yet a considerable number view them as insufficient, underscoring the urgent need for diversification and enhancement of compensation methodologies. Moreover, the differences in preferences for compensation distribution among herders and grassland managers highlight the necessity for a more nuanced legal framework that can accommodate varied stakeholder needs. In addition, this study indicates a strong preference for a shared responsibility model over the government solely bearing the burden of compensation funds. This preference is particularly pronounced in regions with extensive grassland areas, suggesting the limitations of government funding and the need for laws that are adaptable to the practical realities of resource allocation. Furthermore, the research underscores the importance of clear and enforceable property rights as a precondition for efficient and sustainable land use.

This study contributes significantly to the discourse on grassland ecological compensation in China, highlighting the complexity of the issue and the need for legislative reform. It calls for a more flexible and diversified approach to funding and emphasizes the importance of considering the varied preferences and realities of different stakeholders. The study also points to the broader implications for property rights and environmental policy, suggesting a pathway towards more equitable and effective grassland conservation efforts.Please note we have moved the section ‘Ethics approval and consent to participate’ to the end of the methods, as per house style.Thank you very much.

### Supplementary Information

Supplementary Information.

## Data Availability

All data generated or analysed during this study are included in this article and its supplementary information files.

## References

[1] Li D, Xu D, Wang Z, Ding X, Song A (2018). Ecological compensation for desertification control: A review. J. Geog. Sci..

[2] Salzman J, Bennett G, Carroll N, Goldstein A, Jenkins M (2018). The global status and trends of payments for ecosystem services. Nat. Sustain..

[3] Dong Q, Liu X (2020). The legal norms of ecological compensation in coastal cities under regional cooperation. J. Coast. Res..

[4] Wang L, Lv T, Zhang X, Hu H, Cai X (2022). Global research trends and gaps in ecological compensation studies from 1990 to 2020: A scientometric review. J. Nat. Conserv..

[5] Liu D, Chang Q (2015). Ecological security research progress in China. Acta Ecol. Sin..

[6] Gaodi X, Shuyan C, Chunxia L, Changshun Z, Yu X (2015). Ecology, Current status and future trends for eco-compensation in China. J. Resour..

[7] Cao H, Li M, Qin F, Xu Y, Zhang L, Zhang Z (2022). Economic development, fiscal ecological compensation, and ecological environment quality. Int. J. Environ. Res. Public Health.

[8] Oberthür, S., Buck, M., Müller, S., Pfahl, S., Tarasofsky, R. G., Werksman, J. & Palmer, A. J. B. E. Participation of non-governmental organisations in international environmental governance: Legal basis and practical experience. (2002).

[9] Bebbington A, Farrington J, Lewis DJ, Wellard K (2005). Reluctant partners? Non-governmental Organizations, the State and Sustainable Agricultural Development.

[10] Wandesforde-Smith G, Denninger Snyder K, Hart LAJJ (2014). Biodiversity Conservation and Protected Areas in China: Science, Law, and the Obdurate Party-State.

[11] Blicharska M, Hedblom M, Josefsson J, Widenfalk O, Ranius T, Öckinger E, Widenfalk LA (2022). Operationalisation of ecological compensation–Obstacles and ways forward. J. Environ. Manag..

[12] Wang X, Han J, Dong Y (2005). Recent grassland policies in China: An overview. Outlook Agric..

[13] Plummer J, Taylor JG (2013). Community Participation in China: Issues and Processes for Capacity Building.

[14] Li JM, Wang N (2022). How and to what extent is ecosystem services economic valuation used in coastal and marine management in China?. Mar. Policy.

[15] Qu Q, Tsai S-B, Tang M, Xu C, Dong W (2016). Marine ecological environment management based on ecological compensation mechanisms. Sustainability.

[16] Ze H, Wei S, Xiangzheng D (2017). Progress in the research on benefit-sharing and ecological compensation mechanisms for transboundary rivers. J. Resour. Ecol..

[17] Cai W, Liu C, Zhang C, Ma M, Rao W, Li W, Gao M (2018). Developing the ecological compensation criterion of industrial solid waste based on emergy for sustainable development. Energy.

[18] Yildirim K, Ayna YE (2018). The role of non-governmental organizations in environmental governance in Turkey. Adam Acad. J. Soc. Sci..

[19] Chen C, Matzdorf B, Zhen L, Schroeter B (2020). Social-Network Analysis of local governance models for China’s eco-compensation program. Ecosyst. Serv..

[20] Yang W, Gong Q, Zhang X (2020). Surplus or deficit? Quantifying the total ecological compensation of Beijing-Tianjin-Hebei Region. J. Geogr. Sci..

[21] Lewis D, Kanji N, Themudo NS (2020). Non-governmental Organizations and Development.

[22] Zhen H, Qiao Y, Zhao H, Ju X, Zanoli R, Waqas MA, Lun F, Knudsen MT (2022). Developing a conceptual model to quantify eco-compensation based on environmental and economic cost-benefit analysis for promoting the ecologically intensified agriculture. Ecosyst. Serv..

[23] Zhang H, Xu T, Feng C (2022). Does public participation promote environmental efficiency? Evidence from a quasi-natural experiment of environmental information disclosure in China. Energy Econ..

[24] Wang K, Ou M, Wolde Z (2020). Regional differences in ecological compensation for cultivated land protection: An analysis of Chengdu, Sichuan Province, China. Int. J. Environ. Res. Public Health.

[25] Zhao Y, Wu FP, Li F, Chen XN, Xu X, Shao ZY (2021). Ecological compensation standard of trans-boundary river basin based on ecological spillover value: A case study for the Lancang-Mekong River Basin. Int. J. Environ. Res. Public Health.

[26] Li X, Wang Y, Yang R, Zhang L, Zhang Y, Liu Q, Song Z (2022). From “blood transfusion” to “hematopoiesis”: Watershed eco-compensation in China. Environ. Sci. Pollut. Res..

[27] Yu Z, Zhao Q (2022). Research on the coordinated governance mechanism of cross-regional and cross-basin ecological compensation in the Yangtze river delta. Int. J. Environ. Res. Public Health.

[28] Zellweger-Fischer J, Kéry M, Pasinelli G (2011). Population trends of brown hares in Switzerland: the role of land-use and ecological compensation areas. Biol. Conserv..

[29] Shang H, Fan J, Fan B, Su F (2022). Economic effects of ecological compensation policy in Shiyang River Basin: Empirical research based on DID and RDD models. Sustainability.

[30] Martens S (2006). Public participation with Chinese characteristics: Citizen consumers in China’s environmental management. Environ. Polit..

[31] Zhang K, Zongguo W, Liying P (2007). Environmental policies in China: Evolvement, features and evaluation. China Popul. Resour. Environ..

[32] Zhang X, Li F, Li X (2021). Bibliometric analysis of ecological compensation and its application in land resources. Landsc. Ecol. Eng..

[33] Yuanchuan Y, Yukun Z, Jie Z, Si H, Man Z, Long H (2022). An ecological compensation mechanism based on the green productive area of cities. J. Resour. Ecol..

[34] Yu H, Chen C, Shao C (2023). Spatial and temporal changes in ecosystem service driven by ecological compensation in the Xin’an River Basin China. Ecol. Indicat..

[35] Coase RH (1993). Law and economics at Chicago. J. Law Econ..

[36] Tacconi L (2012). Redefining payments for environmental services. Ecol. Econ..

[37] Chen Q, Yu H, Wang Y (2021). Research on modern marine environmental Governance in China: Subject identification, structural characteristics, and operational mechanisms. Int. J. Environ. Res. Public Health.

[38] Zhai T, Wang J, Fang Y, Huang L, Liu J, Zhao CJS (2021). Integrating ecosystem services supply, demand and flow in ecological compensation: A case study of carbon sequestration services. Sustainability.

[39] Shang W, Gong Y, Wang Z, Stewardson MJ (2018). Eco-compensation in China: Theory, practices and suggestions for the future. J. Environ. Manag..

[40] Engel S, Pagiola S, Wunder S (2008). Designing payments for environmental services in theory and practice: An overview of the issues. Ecol. Econ..

[41] Muradian R, Corbera E, Pascual U, Kosoy N, May PH (2010). Reconciling theory and practice: An alternative conceptual framework for understanding payments for environmental services. Ecol. Econ..

[42] Han X, Cao T (2022). Study on the evaluation of ecological compensation effect for environmental pollution loss from energy consumption: Taking Nanjing MV Industrial Park as an example. Environ. Technol. Innov..

[43] Dong, S., Wen, L., Liu, S., Zhang, X., Lassoie, J. P., Yi, S., Li, X., Li, J. & Li, Y. Vulnerability of worldwide pastoralism to global changes and interdisciplinary strategies for sustainable pastoralism. *Ecol. Soc.* **16**(2) (2011).

[44] Yeh ET (2013). The politics of conservation in contemporary rural China. J. Peasant Stud..

[45] Ho P (2001). Who owns China’s land? Policies, property rights and deliberate institutional ambiguity. China Q..

[46] Peterson RA, Cavanaugh JE (2019). Ordered quantile normalization: A semiparametric transformation built for the cross-validation era. J. Appl. Stat..

[47] Liu J, Li S, Ouyang Z, Tam C, Chen X (2008). Ecological and socioeconomic effects of China’s policies for ecosystem services. Proc. Natl. Acad. Sci..

[48] Wang Y, Zhang Z, Chen X (2022). Spatiotemporal change in ecosystem service value in response to land use change in Guizhou Province, southwest China. Ecol. Indicat..

[49] Xu W, Xiao Y, Zhang J, Yang W, Zhang L, Hull V, Wang Z, Zheng H, Liu J, Polasky S (2017). Strengthening protected areas for biodiversity and ecosystem services in China. Proc. Natl. Acad. Sci..

[50] Besley T (1995). Property rights and investment incentives: Theory and evidence from Ghana. J. Polit. Econ..

[51] De Soto H (2000). The Mystery of Capital: Why Capitalism Triumphs in the West and Fails Everywhere Else.

[52] Schlager E, Ostrom E (1992). Property-rights regimes and natural resources: A conceptual analysis. Land Econ..

[53] Boyce JK (1994). Inequality as a cause of environmental degradation. Ecol. Econ..

[54] Martinez-Alier J (2003). The Environmentalism of the Poor: A Study of Ecological Conflicts and Valuation.

