# Supplementary material for: A comprehensive study on non-governmental actors in shaping grassland ecological compensation within legal frameworks
Source: Sci Rep. 2024 Mar 6;14:5489. doi: 10.1038/s41598-024-56146-7 (PMC10917782; doi:10.1038/s41598-024-56146-7)
Supplement: Supplementary file 1 — Supplementary Information. [file 41598_2024_56146_MOESM1_ESM.docx]

**A Comprehensive Study on the Role of Non-Governmental Actors in Shaping Grassland Ecological Compensation within Legal Frameworks**

Liu Ziqi^1*^; SUN Jiyao^2^,

1.Doctoral student；College of Politics and law, Northeast Normal University；Changchun City , Jilin Province，China；liuzq888@nenu.edu.cn；Reseacrh interests: Contemporary Chinese Legal System Construction

2. Associate Professor；School of Marxism，Xidian University；Xi'an City, Shanxi Province, China; sunjiyao@xidian.edu.cn; Reseacrh interests: Comparative ideological and political education

**The Questionnaire**

Dear Respondent：

Greetings from Northeast Normal University research group! We are conducting a study on “The Role of Non-Governmental Subjects in Multiple Ecological Compensation”. Your participation in this study is voluntary and all information collected will solely be used for academic purposes. We assure you that there are no risks to you, your family or your organization. Please find a moment to answer the following questions, thereby contributing to the development of China's grassland ecological compensation legal system.

Best regards,

Northeast Normal University Research Team

Personal Information:

1. Please indicate your age bracket:

A. Under 18 years old

B. Between 18 and 59 years old

C. 60 years old and above

2. What is your level of education?

A. Below primary school

B. Middle school to high school

C. University and above

3. Please indicate your annual net household income range:

A. Less than RMB5,000

B. RMB5,001 to RMB10,000

C. RMB10,001 to RMB30,000

D. More than RMB30,001

4. How large is your pasture land?

A. Less than 100 acres

B. 101 to 300 acres

C. 301 to 500 acres

D. More than 501 acres

5. Please rate the level of degradation of your pasture land:

A. Not degraded

B. Slightly degraded

C. Moderately degraded

D. Severely degraded

1. Considering that China's current grassland ecological compensation practices lack legal constraints, should these practices be legalized?

A. Yes

B. No

C. No opinion

2. In your opinion, is the existing grassland tenure in China clear?

A. Yes

B. No

C. Somewhat

3. Do you consider the information on grassland ecological compensation (time, location, conditions, amount, etc.) in China to be open and transparent?

A. Yes

B. Somewhat

C. No

4. Would you like to participate in the development of grassland ecological compensation policies and legislation?

A. Yes

B. No

5. Who should be the beneficiaries of grassland ecological compensation?

A. Grassland conservation contributors

B. Grassland ecology disruptors

C. Beneficiaries of grassland ecological protection

D. Individuals affected by grassland ecological protection

E. Grassland eco-industry developers

F. All of the above

6. Is the current grassland ecological compensation standard reasonable (i.e., 6 yuan per mu per year for grazing ban, 6 yuan per mu per mu for grass-livestock balance, 1.50 yuan per year)?

A. Generally reasonable

B. Too low, needs to be increased

C. Unclear

7. What should be the basis for determining grassland ecological compensation standards?

A. Value of grassland ecosystem services

B. Contributions from grassland ecological protectors

C. Opportunity costs for grassland ecological protectors

D. Benefits for beneficiaries of grassland ecological protection

E. Cost of restoration of grassland ecological damage

8. Do you find the current methods of compensation (primarily monetary, food and grass seed) reasonable?

A. Generally reasonable

B. Unreasonable, too singular

C. Reasonable, but needs diversification

9. Which grassland ecological compensation approach do you think is most effective?

A. Monetary compensation

B. In-kind subsidies

C. Policy-based

D. Project-based

E. Technical support

F. Industrial support

G. Technical training

10. How should the grassland ecological compensation funds be distributed more effectively?

A. One-time lump-sum payment

B. Periodic rewards and penalties on a floating basis

11. Who should be responsible for providing grassland ecological compensation funds?

A. The Government solely

B. A combination of the government, society, market, and involved individuals

12. Should a special body be established to oversee the enforcement of grassland ecological compensation rights?

A. Yes

B. No

C. No opinion

13. Who should be responsible for providing grassland ecological compensation?

A. Government departments

B. Beneficiaries of grassland ecological protection

C. Destroyers of grassland ecology

D. All of the above

14. How do you evaluate the current obligations (restoration deadlines, fines, etc.) of the parties responsible for grassland ecological compensation (primarily those causing ecological damage)?

A. Generally reasonable

B. Needs more obligations

C. Needs fewer obligations

15. How would you rate the fulfillment of obligations by the parties responsible for grassland ecological compensation?

A. Generally fulfilled

B. Selectively fulfilled

C. Not fulfilled

16. How do you evaluate the obligations of the beneficiaries of grassland ecological compensation (return of pasture to grass, grazing bans, implementation of grass-livestock balance, etc.)?

A. Generally reasonable

B. Needs more obligations

C. Needs fewer obligations

17. How would you rate the fulfillment of grassland protection obligations by the beneficiaries of grassland ecological compensation?

A. Generally fulfilled

B. Selectively fulfilled

C. Not fulfilled
